# Improving hidradenitis suppurativa management: consensus statements from physicians and patients’ perspectives

**DOI:** 10.1007/s00403-024-03316-2

**Published:** 2024-08-24

**Authors:** Angelo Valerio Marzano, Cristina Magnoni, Giuseppe Micali, Angelina De Magnis, Giuseppina Pintori, Simone Fiorini, Valentina Simonella, Linda Bastioli, Francesca Nappi, Giovanni Pappagallo, Francesca Prignano

**Affiliations:** 1 Dermatology Unit, Fondazione IRCCS Ca’ Granda Ospedale Maggiore Policlinico, Via Pace, 9, 20122 Milan, Italy; 2 Department of Pathophysiology and Transplantation, Università Degli Studi Di Milano, Via Pace, 9, 20122 Milan, Italy; 3 Università Degli Studi Di Modena E Reggio Emilia, Azienda Ospedaliero-Universitaria Di Modena, Modena, Italy; 4 Università Di Catania, AOU Policlinico Vittorio Emanuele, Catania, Italy; 5 Azienda Ospedaliero Universitaria Careggi, Florence, Italy; 6 Passion People Aps, Rome, Italy; 7 AISI–Associazione Italiana Sostegno Idrosadenite, Viterbo, Italy; 8 Scuola Di Metodologia Clinica, IRCCS Ospedale Sacro Cuore Don Calabria, Negrar Di Valpolicella, Verona, Italy; 9 Dipartimento Di Scienze Della Salute, Sezione Di Dermatologia, Università Di Firenze, Florence, Italy

**Keywords:** Multidisciplinary approach, HS-unit, Quality of life, Biologics, Patient’s journey

## Abstract

Hidradenitis suppurativa (HS) is a frequent chronic inflammatory skin condition primarily affecting areas rich in apocrine sweat glands, presenting with comedones, papules, nodules, and abscesses, as well as tunnels and hypertrophic scarring. Despite its prevalence and impact on quality of life, the pathogenesis of HS remains incompletely understood. Notably, its diagnosis often suffers from delays and misidentification, therefore an improved education for healthcare providers is mandatory. Moreover, HS is frequently associated with systemic comorbidities and a multidisciplinary approach is suggested for its management. Recognizing these challenges, a group of Italian HS experts and patients convened to develop consensus guidelines via the Nominal Group Technique. Through iterative meetings and remote collaboration, they identified key areas for improvement and formulated consensus statements to guide healthcare providers in delivering optimal care. This collaborative effort highlights the importance of standardized approaches and interdisciplinary collaboration in managing HS effectively.

**Supplementary Information:**

The online version contains supplementary material available at 10.1007/s00403-024-03316-2.

## Introduction

Hidradenitis suppurativa (HS) is a chronic, inflammatory skin disease presenting with comedones, papules, nodules, and abscesses, as well as tunnels and hypertrophic scarring, which occur in areas with a high density of apocrine sweat glands, like skin folds, gluteal and genital sites [1, 2]. Innate immunity dysregulation and proinflammatory cytokines overexpression play a role in HS pathogenesis, which, however, remains not fully clarified [3]. To date, several genetic variants associated to disease susceptibility have been reported [4, 5] but genotype–phenotype correlation and genetic markers predicting the outcome and response to treatment are still lacking. HS is typical for adolescents and young adults but a smaller proportion of individuals develop HS later in life [2]. HS estimated prevalence is 0.05–4.1% worldwide, resulting in a quite common disease [6]. Females are mainly affected, with three-quarters of HS diagnoses being among women, who therefore need specific considerations and management strategies, particularly during pregnancy [7]. HS harms quality of life (QoL) of patients suffering from this condition, which is frequently barely controlled by treatments, leads to chronic pain, reduces mobility and social functioning [8]. Diagnosis is based on clinical criteria (i.e., lesion morphology, distribution of lesions, chronicity, and recurrence) [8], and early identification remains difficult: a European study reported a median delay of 7.2 years [9]. Indeed, HS misdiagnosis is still an issue; therefore, educating healthcare providers (HCPs), including non-dermatologists, would be helpful [10]. HS severity is still most frequently assessed by the Hurley staging systems; however, many score systems are being developed each year but none of which have achieved unanimous acceptance yet [11]. HS is often associated with non-cutaneous comorbidities (e.g., metabolic syndrome, obesity, arthritis) and has to be considered a systemic disease [12–16]. For instance, data from HS patient Italian registry show that HS adults are more frequently overweight or obese than the general Italian population (age-standardized prevalence ratio of 1.4; 95% CI, 1.1–1.9) [17]. Considering clinical manifestations complexity and variety, HS requires in-depth evaluations and an appropriate referral to specialists [12]. In this context, multidisciplinary clinics (defined as HS units), where different specialists (like dermatologists, surgeons, nutritionists, and psychologists) collaborate, would guarantee additional support to patients. However, such clinics are still very rare [10]. HS treatment is based on a multidisciplinary approach including medical and surgical treatments, together with general lifestyle recommendations [18]. As therapies are complex and often ineffective, improving treatment options, including both drugs and surgery, remains an unmet need [10].

To report and address the current unmet needs related to HS patient management, five Italian HS experts and five patients met in person twice. Their primary objective of these gatherings was to elaborate a consensus document to provide HCPs with standardized guidance to achieve appropriate HS management, focusing on patients’ wellbeing. To this purpose, experts and patients identified 14 relevant items based on published evidence and personal experience and formulated a series of consensus statements for each item. Consensus was reached using the Nominal Group Technique (NGT), with the involvement of an expert panel, including a methodologist. Here, we present the results of this effort.

## Methods

Five Italian HS experts, five patients, and one senior clinical epidemiologist experienced in consensus techniques (*facilitator*) took part in two in-person meetings occurring in December 2023 and January 2024 to address issues in the management of HS and achieved a consensus paper. To identify topics of interest (*items*), NGT was selected as the first formal consensus approach. NGT aims to avoid typical problems generated from group dynamics, enhancing the independent and free participation of each member of the group, who can express his or her ideas without restrictions [19]. NGT historically consists of four main stages: silent generation, “round robin”, clarification, and voting. These steps are followed by a final group discussion of the items and a re-ranking process till the achievement of the consensus [20]. During this meeting, participants were initially asked to answer a question about the main aspects of interest and unmet needs in HS patient management, including the early approach to them. After reflecting on topics, they wrote down their ideas (*idea generation*), aware that their written opinions would not be editable. After this initial stage, the facilitator asked the participants to state their views, one at a time (*round robin*); then, all the members of the group discussed (*clarification*) and ranked the collected ideas (*ranking of items*). To generate statements concerning each of agreed item, the estimate-talk-estimate (ETE) method was chosen [21, 22]. ETE was again developed to overcome some of the negative aspects of group dynamics by combining the assembling of expert opinions on an anonymous basis during surveys with an open exchange during workshops [23–25]. Thus, the board members individually drew up one or more statements for each of the 14 items. The proposals were remotely harmonized by the facilitator into 50 statements. During the second in-person meeting, the board members and the facilitator reviewed and further discussed the harmonized statements, and finally agreed on a total of 52 statements, grouped by subject, and presented here as a consensus.

## Results and discussions

The consensus statements covering relevant issues about HS patient management are shown in Table 1*(Suppl. Material)*. The statements were collected and grouped into 14 main sections (or items) by topic and discussed with the evidence supporting them.

### Implementation of a common approach in the management of HS, through the spread of a solid and consistent knowledge of the disease

1.1 HS is a skin disease not fully recognized by physicians, while it is deeply known by specialists working in referral centers, where patients enter with delay after many visits. This leads to delayed diagnosis and treatment start. In-depth knowledge about HS has to be spread across the nation.
1.2 Widespread knowledge of HS is a priority that has to be achieved through different communication strategies, including traditional (television, newspapers, radio) and new online media (social media) along with collaboration with patients’ associations.
1.3 A standard approach to HS all across the nation is fundamental and consisting of common strategies between and within regions. This will allow an appropriate understanding of the disease (among physicians and patients) and the access to adequate care for patients.
1.4 Scientific societies and political institutions should develop awareness about HS.
  1.4.1 The task force established by SIDeMaST (Società Italiana di Dermatologia e di Malattie Sessualmente Trasmesse) together with other experts’ networks should train dedicated specialists (*frontline HCPs*).
  1.4.2 Institutions should ensure the presence of HS units, in referral dermatologic clinics, where patients can be referred by *frontline HCPs* working in local clinics, through an efficient network (hub and spoke model).
1.5 There is need for defining the criteria that identify dermatologic referral centers (HUBs) for the treatment of HS involving patient associations in their definition, as provided for by EU Regulation 2021/2282, and for mapping these centers in each region of the nation.

#### Rationale

Awareness on HS is still low among physicians, although it is not a rare disease (around 1% of global population is affected). All physicians taking care of patients with confirmed or suspected HS, should be efficiently informed and trained. Thus, specific knowledge should be disseminated also through non-dermatologic channels (both conferences and journals) [26]. A unified HS management is thus necessary nationwide, with the establishment and dissemination of HS units playing a crucial role in this regard. HS-units represent a model of multidisciplinary approach based on several HCPs involvement [27]. This will help patients to get a standardized clinical approach, and an equal and fair access to care. Moreover, general practitioners and local dermatologists are pivotal within this network as frontline healthcare providers, tasked with early disease recognition and prompt patient referral to HS units. Collaboration with these units is essential [28]. Training frontline HCPs is necessary to achieve this goal.

### Phenotypes diagnosis and severity assessment of HS

2.1 An accurate diagnosis of the multiple phenotypes of HS is crucial. The most challenging aspect of the diagnostic process is due to the several “unusual” phenotypes of the disease other than the “classic” one, which can lead to misdiagnoses and require different therapeutical approaches.
2.2 The implementation of a scoring system to determine HS severity is a cornerstone of the management and treatment of this condition.
2.3 The IHS4 (International Hidradenitis Suppurativa Severity Score System) is a novel scoring system, build by the European HS Foundation, that assesses both inflammatory (nodules and abscesses) and advanced (fistulas) lesions. The IHS4-55 is an updated dichotomous version able to evaluate responses to treatment, taking into account not only inflammatory, but also advanced lesions, like HiSCR (Hidradenitis Suppurativa Clinical Response) score -the most common primary outcome used in HS clinical trial- does.

#### Rationale

Given the absence of a specific diagnostic test, HS is diagnosed based on clinical evaluation [29]. The most updated and validated classification include up to 6 HS phenotypes: (1) regular type; (2) frictional furuncle type; (3) scarring folliculitis type; (4) conglobate type; (5) syndromic type; (6) ectopic type [29]. Considering that knowledge of underlying HS pathogenetic mechanisms is still lacking, phenotypic variants could be revised in the future [30]. Physicians have to take into account differential diagnoses (e.g., infections, tumors) when suspecting HS, being aware of the variety of involved sites and of atypical lesions that characterize HS (e.g., eroded pyogenic granuloma-like lesions), also contributing to misdiagnoses [26, 31]. Clinical manifestations variability requires an in-depth expertise in HS to formulate an accurate diagnosis. Many HCPs, whilst diagnosing HS, are not able to evaluate disease severity [28]. Although Hurley scoring system remains the most known and used classification to stratify HS severity, it displays limitations: it includes only 3 stages and is not useful for monitoring the efficacy of a treatment [29]. With the advent of novel effective therapies, new score systems were developed to differentiate clinical therapeutic responses. The HiSCR, defined in 2012, identifies treatment responders as those who achieve at least a 50% reduction in abscess and nodule count, without an increase in the number of abscesses or draining tunnels relative to baseline [30]. In 2017 the IHS4 was created as a continuous score combining inflammatory nodules, abscesses and draining tunnels [32]. A threshold application (i.e., 55% reduction of the IHS4 total score) has allowed IHS4-55 definition, a novel dichotomous IHS4 version that may rival HiSCR as a primary outcome measure in HS clinical trials [33].

### Collaboration between patients’ associations and the scientific community to promote social protection for HS patients

3.1 Complex and chronic diseases, such as HS, should be handled coordinately and a multidisciplinary approach represents the most effective strategy for managing these patients.
3.2 Patients’ associations should be involved in the multidisciplinary teams that approach HS patients, with particular regard to organization activities and relationships with the institutions for better focusing on the unmet needs.
3.3 Collaboration between patients’ associations and the scientific community represents a cornerstone for ensuring equity in providing timely access to diagnostic tools and innovative treatments, and social welfare for patients: recognition of HS as a chronic and disabling condition, leading to the right to full exemption, recognition of disability and handicap for adults and minors, smart working for fragile patients, and activation of distance learning for students with HS. Moreover, Essential Levels of Care (LEA) require a detailed and comprehensive update that will consider the needs of patients diagnosed with HS.

#### Rationale

Currently, an integrated multidisciplinary model is considered the best strategy to manage HS patients, reporting a high satisfaction level with this approach [27, 28, 34]. HS determines a profound QoL impairment due to mental disturbances, such as depression or anxiety, [35], comorbidities [12] and feelings of shame and stigmatization [26]. Patients often feel themselves responsible for their condition and do not seek medical care [26]. Evidence shows that HS has a severe impact on QoL in 60% of patients [36]. For these reasons, the disease management is moving toward an holistic and patient-centered approach [37] where patients’ associations should collaborate with multidisciplinary teams and scientific community to guarantee targeted interventions. Panelists suggest remote education for students, smart working, and psychological support since diagnosis as interventions to promote social protection to these patients.

### Improvement of the patient journey

4.1 Improvement of the diagnostic and therapeutic path for HS patients is a central goal. In this context, all the figures involved in the management of this condition–general practitioners, local dermatologists, triage nurses working in departments of emergency and admission (DEA) are involved—need specific education to improve their disease awareness, appropriately manage patients, and facilitate patients access to HS-dedicated centers.
4.2 The creation of a common diagnostic and therapeutic care pathway (Percorso Diagnostico-Terapeutico Assistenziale, PDTA) reflects a specific operating model of a multidisciplinary team, that would guarantee standardized activities, risk identification, and transition of care. If this model will be applied to a regional level, it could support patients in accessing the centers included in the PDTA.

#### Rationale

HS patients may experience a very long and chaotic “journey” to achieve a correct diagnosis and start an effective treatment. This non-linear “journey” may include numerous visits with different physicians determining a diagnostic delay between 6 and 10 years, according to most studies [26, 28, 38]. Delayed HS diagnosis remains a barrier and the latest studies did not show any shortening in diagnosis timing [39]. Patients often search for medical information online before seeking for medical evaluation, and even after the first medical consultation, the patient may experience “ineffective” visits before achieving a correct diagnosis [38]. Moreover, after a patient receives a definite HS diagnosis, the beginning of an effective treatment may be delayed due to the low expertise in the HS management among the HCPs who first see these patients [38]. Patients should be involved as central figures in this pathway, which should be not only clinical, but also informative and emotional for them. To inform patients, organizations play a key role, since after diagnosis, patients typically contact those to get support [38]. An improvement of the patient journey would impact various aspects, such as diagnosis, disease severity assessment, and treatment approach. To overcome this barrier, a multidisciplinary model has been proposed, with the involvement of multiple HCPs with specific roles, who interact together to facilitate the patient journey toward and within HS-dedicated units [28].

### Main identified unmet needs

5.1 Currently, only few centers can provide all the surgical procedures for HS, including minor and major demolitive (i.e., wide surgical excision) procedures. Furthermore, across Italy, a lack of disease awareness among general and plastic surgeons causes non-adequate priority to patients suffering from HS.
5.2 Long-term hospitalizations are frequently necessary after wide surgical excisions due to the possible occurrence of several complications, such as infections.
5.3 Centers able to accurately diagnose HS are widely needed.
  5.3.1 Centers for HS diagnosis should be equipped with advanced and targeted diagnostic instruments, such as high frequency ultrasound (HFUS), magnetic resonance imaging (MRI), and medical infrared thermography (MIT).
  5.3.2 Fundings should be budgeted for providing centers with basic diagnostic instruments in order to fill the current gaps in the management of HS.
  5.3.3 It is of primary importance to reduce waiting lists for specialist appointments within the referral dermatological centers for HS to ensure optimal management and monitoring of the condition.

#### Rationale

HS surgical approaches include many options [8, 40]. There is evidence that post-surgical complications are quite common. A metanalysis (including 13 studies and 535 patients) reported an average estimated complication rate of 11.1%, but showed at the same time that surgical resection represents perhaps the most effective treatment of severe and advanced HS [40]. Higher long-term post-surgical complication rates have been observed, even if in only one study [41]. There is a clear need for specialized centers for HS-related surgery, where surgeons should work with standardized surgical approaches and techniques, while increasing their awareness of this condition [40]. An improvement of surgical options represents an unmet need, according to patients diagnosed with HS [10]. The “Operational Core” team of the multidisciplinary model proposed by many HS experts comprises a radiologist [27], who has a central role because the diagnostic process of HS includes imaging techniques, which often allow to detect minimal/mild presentations [42]. Both MRI and US can improve disease severity and the extent of the evaluation, facilitating HS management [43]. MRI plays also a specific role in assessing severe anogenital lesions [43, 44]. MIT is a non-invasive diagnostic test assessing inflammation of HS lesions, by measuring real-time temperature. It represents a promising diagnostic approach, in combination with US and MRI, for HS diagnosing and staging [45]. Access to centers equipped with such advanced and reliable diagnostic tools should be ensured for all patients with suspected or confirmed HS. It is of primary importance to reduce waiting lists for specialist appointments within the referral dermatological centers for HS to ensure optimal management and monitoring of the condition.

### Definition and characteristics of HS-dedicated units: the importance of a multidisciplinary approach for a disease with cutaneous and extra-cutaneous manifestations

6.1 HS is linked with a high burden of comorbidities, including non-cutaneous manifestations. Comorbidities associated with HS are metabolic, cardiovascular, endocrinological, gastrointestinal, rheumatological, and psychiatric diseases. All of these negatively affect patients’ QoL.
6.2 Definition of the HS-unit is an essential step toward an optimal treatment of patients suffering from such a complex disease. A multidisciplinary approach is the key to guarantee a comprehensive management of these patients, taking into consideration all the aspects related to the disease.
6.3 The HS-unit should be part of a dermatologic referral center. A dermatologist with specific expertise in HS should coordinate the unit and work with a team including dermatologists, plastic or general surgeons, wound care specialists and nurses with expertise in HS within the unit.
6.4 In HS patients, the development of different kinds of wounds (not only post-surgical) is a common situation that necessitates the involvement and intervention of wound care specialists. In this context, specific training for wound care nurses represents a priority.
6.5 Based on the most frequent comorbidities associated with HS, the multidisciplinary team working in the HS-unit should include an infectious diseases specialist, a pain specialist, a gynecologist, a urologist, an endocrinologist, a rheumatologist, a gastroenterologist, a cardiologist, an andrologist, a nutritionist, a psychiatrist or a psychologist, a pneumologist.
6.6 Rapid access to the HS-unit should be guaranteed to patients with suspected HS and a family history of HS.
6.7 Medical visits should have an appropriate duration to allow a precise and in-depth evaluation of physical and psychological conditions of referred patients. This achievement is crucial for obtaining a definite diagnosis and a personalized treatment.

#### Rationale

HS is a chronic condition, that does not only affect the skin, but also relates to many extracutaneous comorbidities. People with HS have a twofold increased risk of developing Crohn’s disease and a 1.5-fold increased risk of ulcerative colitis, and tend to be at higher risk of metabolic syndrome (i.e., obesity, hypertension, dyslipidemia, diabetes) compared to the general population [8, 12]. Inflammatory arthritis is more common among HS patients [8]. Psychiatric conditions, like depression and anxiety, are common in these patients with a prevalence of 16.9% and 4.9%, respectively [8, 12]. The HS multidisciplinary units are organized systems able to manage patients suffering from such a complex disease presenting both cutaneous and extracutaneous manifestations. The main Operational Core of the unit should include four HCPs: a dermatologist, a surgeon, a radiologist, and a nurse/wound care specialist, responsible for the medical and surgical treatments of HS patients. In support of these figures, a panel of consultants should participate in the management of the comorbidities; among the others, gastroenterologists, endocrinologists, gynecologists, urologists/andrologists, rheumatologists, psychiatrists, cardiologists, infectious diseases and pain specialists, should step in, when required by the Operational Core [27, 28]. Moreover, local professionals (i.e., general practitioners and local dermatologists) should be integrated, as frontline care providers, in the network to facilitate the access of patients to HS-units [27, 28, 38]. Physicians visiting patients diagnosed with HS should have an adequate amount of time to evaluate appropriately each of them, in order to face also the psychological burden endured by patients. Since many genetic variants have been associated with HS predisposition, patients with a HS family history and any diagnostic criteria for HS diagnosis should access HS-clinics with a certain grade of priority [46, 47].

### Personalization of therapy according to patient’s characteristics

7.1 To provide an optimal treatment, individual characteristics should be considered, including: age, gender, pre-existent diseases, life-style, HS duration and previous therapies.
7.2 Therapeutic approach differs between children and adults and current available treatments should be carefully evaluated given the lack of specific guidelines to date.
7.3 A pediatric patient could not tolerate a specific treatment, while accepting a different but equally effective one.
7.4 Pregnant women require a specific management of the disease, they should be evaluated more closely than general population due to possible complications related to pregnancy and risks related to some pharmacological therapies.
7.5 Among women of childbearing age, hormonal therapies should be considered, in agreement with gynecologists. For instance, estrogen/progestin treatments to prevent HS flares during premenstrual period.
7.6 HS should be considered a disease having high priority among those taken into account by the National Observatory for Gender Medicine.

#### Rationale

HS therapeutic options currently range from topical and oral antibiotics to biologic agents and immunomodulators [48], in combination or not with surgical treatments [49]. A personalized therapy, based on the specific HS patient characteristics, has been suggested by The European S1 HS guideline [49]. The HS-units integrated multidisciplinary care model aims to offer a personalized treatment to patients, achieving higher efficacy, compliance and satisfaction [34]. First of all, age is a factor to consider when clinicians approach HS patients. This disease is quite uncommon in elderly people where HS is associated with delayed wound healing and infection occurrence during biologic therapy [50]. In a cohort of 26 patients aged 65 and older, HS was more likely associated with comorbidities and Hurley 3 stage at diagnosis [50]. Scientific literature about HS in pediatric population is limited. HS diagnosis is often delayed in children, who typically present somatic and psychiatric comorbidities and complications (e.g., scarring) when diagnosed with HS [51]. Medical treatment is challenging due to lack of clinical research and data, with very few options among immunomodulators. There is evidence that most of them are treated with topical or systemic antibiotics [51]. Gender differences are identified in HS epidemiology and clinical course, with a female-to-male ratio in prevalence of 3:1 (in Europe and North America) and different cutaneous features in women [52, 53]. Hormones play a role in the course of the disease, although which one is not yet clarified [7, 54]. Women with HS should receive a specific care, in particular if pregnant [7] since they could experience a HS worsening during pregnancy. A study accounting for 202 pregnancies, showed that HS worsened in 61.9% cases [55]. However, HS deterioration and changes in hormone levels in pregnancy are not consistent among all women and further clarifications are still needed [53]. Dermatologists should manage these patients together with gynecologists [55]. More than half of the women report HS peri-menstruation flare-ups [53] and there is limited evidence that estro/progestins may help in these cases [54].

### Education on HS of health-care providers taking care of the patients (including clinicians, nurses, psychologists, nutritionists, surgeons, and case managers)

8.1 Considering the complexity of this disease with such a strong existential impact, HCPs should be specifically trained, qualified and motivated.
8.2 A HS disability manager from patients’ associations working in the training teams could have a positive impact on the education of HCPs.

#### Rationale

Beyond physical impact, HS patients suffer also from profound psychological disorders [48]. A study evaluating 38 HS patients through psychometric questionnaires showed that they experience lower self-esteem, higher levels of anger and of emotional fragility rather than a control group [56]. Therefore, the panelists agreed that continuing medical education on HS is essential for HCPs taking care of these patients. Medical doctors should be trained on this topic starting from their medical school and then during residency program in Dermatology. Moreover, knowledge and awareness about HS-related pain has been reported as inadequate among HCPs, although patients report pain as the most relevant symptom. Education of all specialists about this topic would ameliorate pain management and patients’ QoL [57]. Clinicians working within HS-units should attend periodical meetings for sharing the most up-to-date knowledge and their experiences.

### Humanization of care and holistic approach for patients diagnosed with HS

9.1 Management of HS patients should be humanly and psychologically appropriate: HCPs should be perceived by the patients as sympathetic allies understanding their problems.
9.2 Communication should be bilaterally complete, with proper time for listening and comprehensive answers to obtain a global understanding of all the problems related to the disease.
9.3 Involving families and caregivers can help patients accept the complex diagnostic and therapeutic pathways, also reassuring them and increasing their confidence in HCPs.
9.4 HCPs should develop new specific skills, as well as novel healthcare management strategies considering to create all activities related to humanization of care.
9.5 The goal is to support patients diagnosed with HS throughout a shared path, characterized by clear information, respect of time for listening, clinically appropriate answers, in an environment that patients perceive as empathetic.

#### Rationale

After HS diagnosis, patients try to live their lives despite this debilitating disease [38]. Panelists agreed that patient education together with a clear communication between doctors and patients can give patients instruments to enhance disease awareness. Understanding patients’ perspective is critical for optimal management: a qualitative study with 3 focus groups analyzing the experiences of HS patients, highlighted their need for increasing psychological support due to the devastating HS effects on mental health [10]. Even people living with HS patients often experience psychosocial issues, with no tools to support them. Włodarek et al. indicated a moderate impairment on caregivers QoL, correlated with HS severity [58]. Clinicians caring for these patients should respectfully inquire about their opinions when proposing treatment options and collaborate with them to determine the most effective therapeutic approach. This attitude enforces mutual confidence [59].

### Patient-reported-outcomes (PROs) specific for HS patients

10.1 *Ad hoc* PROs assessments should be adopted systematically for all HS patients because they can accurately capture these patients’ lives, considering the huge impact of the disease on QoL. To date, Hidradenitis Suppurativa quality of life (HiSQoL) and Pain Index are the most validated and supported by scientific evidence.

#### Rationale

Although clinicians show a growing interest in the field, validated instruments to assess PROs of HS patients are still scarce, with consequently limited knowledge about the QoL of these patients so far [60]. HiSQoL questionnaire (validated in 2020) contains 17 items, both HS-specific and non-specific [61]. Among the scales evaluating pain, the Pain Index is a recently validated and useful score, assessing both pain intensity and duration [62].

### Paucity of effective treatments currently available for HS to achieve optimal clinical outcomes and improvements in QoL

11.1 Effects of current therapies often do not fulfil clinicians’ and patients’ needs. Therefore, promotion and support of research for new drugs development (with clinical trials) and for optimizing existing treatments (with real-life studies) are fundamental. This would offer new hopes and solutions to patients.

#### Rationale

HS medical treatment includes many drugs (e.g., steroids, antibiotics); among them biologics/immunotherapies represent the most effective options based on current evidence [18, 30, 63]. Three biologics are approved for HS: adalimumab, an antibody against tumor necrosis factor α, studied in PIONEER I and II trials [64], secukinumab, a monoclonal antibody selectively neutralizing IL-17A, evaluated in SUNRISE and SUNSHINE trials [65], and bimekizumab, a monoclonal antibody targeting both IL-17A and F [66]. Adalimumab is currently the only biologic refunded by the Italian Health Service for HS, with most studies evaluating novel treatments being still ongoing with only modest reported clinical efficacy [30]. Panelists suggested the design of further clinical studies with larger collaborations among different specialists to collect data.

### Specific management of flares in HS

12.1 A unanimous approach for the management of flares is not proposed by European and American guidelines currently available. The approach to flares depends on the expertise of dermatologists or surgeons, who treat the patient depending on his/her characteristics and ongoing therapies.
12.2 A clear and common definition of HS flare and bacterial superinfection is necessary. In case of infection a targeted antibiotic therapy should be administered.
12.3 In case of mild HS not being treated with systemic antibiotics, systemic antibiotics (e.g., tetracycline or clindamycin) can be started when flares occur.
12.4 In case of moderate-severe HS being treated with systemic antibiotics, introduction of biologics can be considered when flares occur. Adalimumab is currently the only biologic refunded by the Italian Health Service for HS.
12.5 In case of patients already on biological therapy, an increase in dose or frequency of administration (even temporary) could be considered when flares occur, although this approach (i.e., adalimumab 80 mg weekly) is now off-label.
12.6 In case of frequent, long-lasting or non-responsive to aforementioned therapy flares, a switch to a different biologic drug could be considered, although most of them are currently off-label for HS.
12.7 In case of localized flare in a single site, topical treatments could be considered, both intralesional infiltration of triamcinolone and local surgery, such as deroofing of abscessed lesions.

#### Rationale

Although more than 80% of HS patients suffer from periodic flares [67] neither the European nor American Guidelines specify definition and management of flares [49, 68]. That results in the absence of a reliable recommended approach to patients experiencing flares in current clinical practice. A review on this topic, including 154 studies, highlighted the tangible need for a specific and measurable definition of “HS flare” [69]. In 2022, an international consensus proposed a definition of HS flare as a “new or substantial worsening of clinical signs *or* symptoms” [70]. In this context, panelists summarized here some possible therapeutic options for patients experiencing a flare in different clinical scenarios.

### Combination of medical and surgical treatments for HS

13.1 Surgery is fundamental for specific HS-related conditions, such as abscesses, fistulas or scar tissue removal. A combined approach with specific drugs, such as biologics, is needed to control inflammation, enhancing benefits and reducing the risk of recurrency.

#### Rationale

A multidisciplinary approach is currently recommended for the management of HS, involving different specialists, with dermatologists and surgeons as key players [27, 28]. HS complexity results in the necessity for a combination of medical and surgical treatments, to control inflammation by a double approach [71, 72].

### Smoking in HS patients

14.1 The smoking habit represents a negative prognostic and therapy-response predictive factor. In addition, smoking is a well-known cardiovascular risk factor.
14.2 Active and former smokers could have restrictions to access a novel class of drugs now under study for HS (i.e. JAK-inhibitors).
14.3 The smoking habit should be discouraged in these patients, by enhancing the awareness about smoking consequences in HS and by joining stop smoking support programs.

#### Rationale

The majority of HS patients smoke tobacco [73, 74]. A meta-analysis revealed that HS patients are 4 times more likely to be smokers than controls without HS [75]. Disease severity has been associated with smoking habit, with remission rates being lower among active smokers than nonsmokers (29% vs 40%) [72]. Despite the confirmed role of smoking as a proinflammatory mediator and as an important trigger factor of the disease [52], a clear pathogenetic link to HS occurrence has not been found yet [18]. Considering also HS-related comorbidities (such as cardiovascular disease), smoking cessation should be part of the management of HS patients [30, 76].

## Conclusions

This consensus addresses most of the topics related to HS patient management, highlighting issues and unmet needs in clinical practice for specialists taking care of these patients. Statements, agreed by the group of experts and patients, analyzed the most relevant gaps that not only dermatologists, but all players taking part in the disease multidisciplinary approach, face on a daily basis. The effort of this consensus will hopefully result in providing practical instruments to better manage patients and ensure an improved QoL. Moreover, this may trigger the conception and development of new clinical studies on HS, a disease requiring specific awareness, knowledge, and care.

## Supplementary Information

Below is the link to the electronic supplementary material.

Supplementary file1 (DOCX 23 KB)

## Data Availability

No datasets were generated or analysed during the current study.
