# Supplementary material for: Improving hidradenitis suppurativa management: consensus statements from physicians and patients’ perspectives
Source: Arch Dermatol Res. 2024 Aug 24;316(8):577. doi: 10.1007/s00403-024-03316-2 (PMC11344722; doi:10.1007/s00403-024-03316-2)
Supplement: Supplementary file 1 — Supplementary file1 (DOCX 23 KB) [file 403_2024_3316_MOESM1_ESM.docx]

**IMPROVING HIDRADENITIS SUPPURATIVA MANAGEMENT: CONSENSUS STATEMENTS FROM PHYSICIANS AND PATIENTS’ PERSPECTIVES.**

*Corresponding Author: Angelo Valerio Marzano*

**SUPPLEMENTARY MATERIAL**

**Table 1**. Statements of the consensus.

| ***1. Implementation of a common approach in the management of HS, through the spread of a solid and consistent knowledge of the disease*** |
| --- |
| 1.1 HS is a skin disease not fully known by physicians. HS is better known by specialists working in referral centers, where patients enter with delay after many visits. This leads to delays in diagnosis and treatment start. In-depth knowledge about HS has to be spread across the nation. |
| 1.2 Widespread knowledge of HS is a priority that has to be achieved through different communication strategies, including traditional (television, newspapers, radio) and new online media (social media) along with collaboration with patients’ associations. |
| 1.3 A universal approach to HS all across the nation is fundamental and consisting of common strategies between and within regions. This will allow an appropriate understanding of the disease (among physicians and patients) and the access to adequate care for patients. |
| 1.4 Scientific societies and political institutions should develop awareness about HS.  1.4.1 The task force established by SIDeMaST (Società Italiana di Dermatologia a di Malattie Sessualmente Trasmesse) together with other experts’ networks should train dedicated specialists (*frontline healthcare providers*).  1.4.2 Institutions should ensure the presence of HS units, in referral Dermatologic clinics, where patients can be referred by *frontline providers* working in local clinics, through an efficient network (hub and spoke model). |
| 1.5 There is need for defining the criteria that identify dermatologic referral centers for the treatment of HS involving patient associations in their definition, as provided for by EU Regulation 2021/2282, and for mapping these centers in each region of the nation. |
| ***2. Phenotypes diagnosis and severity assessment of HS*** |
| 2.1 An accurate diagnosis of the multiple phenotypes of HS is crucial. The most challenging aspect of the diagnostic process is due to the several “unusual” phenotypes of the disease, which can lead to misdiagnoses and require different therapeutical approaches. |
| 2.2 The implementation of a scoring system to determine the severity of HS is a cornerstone of the management and treatment of this condition. |
| 2.3 The IHS4 (International Hidradenitis Suppurativa Severity Score System) is a novel scoring system, build by the European HS Foundation, that assesses both inflammatory (nodules and abscesses) and advanced (fistulas) lesions. The IHS4-55 is an updated dichotomous version able to evaluate responses to treatment, taking into account not only inflammatory, but also advanced lesions, like HiSCR (Hidradenitis Suppurativa Clinical Response) score -the most common primary outcome used in HS clinical trial- does. |
| ***3. Collaboration between patients’ associations and the scientific community to promote social protection for HS patients*** |
| 3.1 Complex and chronic diseases, such as HS, should be handled coordinately and a multidisciplinary approach represents the most effective strategy for managing these patients. |
| 3.2 Patients’ associations should be involved in the multidisciplinary teams that approach HS patients, with particular regard to organization activities and relationships with the institutions for better focusing on the unmet needs. |
| 3.3 Collaboration between patients’ associations and the scientific community represents a cornerstone for ensuring equity in providing timely access to diagnostic tools and innovative treatments, and social welfare for patients: recognition of HS as a chronic and disabling condition, leading to the right to full exemption, recognition of disability and handicap for adults and minors, smart working for fragile patients, and activation of distance learning for students with HS.  Moreover, Essential Levels of Care (LEA) would strongly require a detailed and comprehensive update that will consider the needs of patients diagnosed with HS. |
| ***4. Improvement of the patient journey*** |
| 4.1 Improvement of the diagnostic and therapeutic path for HS patients is a central goal. In this context, all the figures involved in the management of this condition – general practitioners, local dermatologists, triage nurses working in departments of emergency and admission (DEA) are involved - need specific education to improve their disease awareness, appropriately manage HS patients, and facilitate patients access to HS-dedicated centers. |
| 4.2 The creation of a common diagnostic and therapeutic care pathway (PDTA) reflects a specific operating model of a multidisciplinary team, that would guarantee standardized activities, risk identification, and transition of care. If this model is applied to a regional level, it could support patients in accessing the centers included in the PDTA. |
| ***5. Main identified unmet needs*** |
| 5.1 Currently, only few centers can provide all the surgical procedures for HS, including minor and major demolitive (i.e., wide surgical excision) procedures. Furthermore, across Italy, a lack of disease awareness among general and plastic surgeons causes non-adequate priority to patients suffering from HS. |
| 5.2 Long-term hospitalizations are frequently necessary after wide surgical excisions due to the possible occurrence of several complications, such as infections. |
| 5.3 Centers able to accurately diagnose HS are widely needed. |
| 5.3.1 Centers for HS diagnosis should be equipped with advanced and targeted diagnostic instruments, such as high frequency ultrasound (HFUS), magnetic resonance imaging (MRI), and medical infrared thermography (MIT). |
| 5.3.2 Fundings should be budgeted for providing centers with basic diagnostic instruments in order to fill the current gaps in the management of HS. |
| 5.3.3 It is of primary importance to reduce waiting lists for specialist appointments within the referral dermatological centers for HS to ensure optimal management and monitoring of the condition. |
| ***6. Definition and characteristics of HS-dedicated units: the importance of a multidisciplinary approach for a disease with cutaneous and extra-cutaneous manifestations*** |
| 6.1 HS is linked with a high burden of comorbidities, including non-cutaneous manifestations. Comorbidities associated with HS are metabolic, cardiovascular, endocrinological, gastrointestinal, rheumatological, and psychiatric diseases. All of these negatively affect patients’ QoL. |
| 6.2 Definition of the HS-unit is an essential step toward an optimal treatment of patients suffering from such a complex disease. A multidisciplinary approach is the key to guarantee a comprehensive management of these patients, taking into consideration all the aspects related to the disease. |
| 6.3 The HS-unit should be part of a dermatologic referral center. A dermatologist with specific expertise in HS should coordinate the unit and work with a team including dermatologists, plastic or general surgeons, wound care specialists and nurses with expertise in HS within the unit. |
| 6.4 In HS patients, the development of different kinds of wounds (not only post-surgical) is a common situation that necessitates the involvement and intervention of wound care specialists. In this context, specific training for wound care nurses represents a priority. |
| 6.5 Based on the most frequent comorbidities associated with HS, the multidisciplinary team working in the HS-unit should include an infectious diseases specialist, a pain specialist, a gynecologist, a urologist, an endocrinologist, a rheumatologist, a gastroenterologist, a cardiologist, an andrologist, a nutritionist, a psychiatrist or a psychologist, a pneumologist. |
| 6.6 Rapid access to the HS-unit should be guaranteed to patients with suspected HS and family history of HS. |
| 6.7 Medical visits should have an appropriate duration to allow a precise and in-depth evaluation of physical and psychological conditions of referred patients. This achievement is crucial for obtaining a definite diagnosis and a personalized treatment. |
| ***7. Personalization of therapy according to patient’s characteristics*** |
| 7.1 To provide an optimal treatment, individual characteristics should be considered, including: age, gender, pre-existent diseases, life-style, HS duration and previous therapies |
| 7.2 Therapeutic approach differs between children and adults and current available treatments should be carefully evaluated given the lack of specific guidelines to date. |
| 7.3 A pediatric patient could not tolerate a specific treatment while accepting a different but equally effective one. |
| 7.4 Pregnant women require a specific management of the disease, they should be evaluated more closely than general population due to possible complications related to pregnancy and risks related to some pharmacological therapies. |
| 7.5 Among women of childbearing age, hormonal therapies should be considered, in agreement with gynecologists. For instance, estrogen/progestin treatments to prevent HS flares during premenstrual period. |
| 7.6 HS should be considered a disease having high priority among those taken into account by the National Observatory for Gender Medicine. |
| ***8. Education on HS of health-care providers taking care of the patients (including clinicians, nurses, psychologists, nutritionists, surgeons, and case managers)*** |
| 8.1 Considering the complexity of this disease with such a strong existential impact, HCPs should be specifically trained, qualified, and motivated. |
| - 1. A HS disability manager from patients’ associations working in the training teams could have a   positive impact on the education of HCPs. |
| ***9. Humanization of care and holistic approach for patients diagnosed with HS*** |
| 9.1 The management of HS patients should be humanly and psychologically appropriate: HCPs should be perceived by the patients as sympathetic allies understanding their problems. |
| 9.2 Communication should be bilaterally complete, with proper time for listening and comprehensive answers to obtain a global understanding of all the problems related to the disease. |
| 9.3 Involving families and caregivers can help patients accept the complex diagnostic and therapeutic pathways, also reassuring them and increasing their confidence in HCPs.  9.4 HCPs should develop new specific skills, as well as a novel strategy of healthcare management considering all the activities related to humanization of care should be build. |
| 9.5 The goal is to support patients diagnosed with HS throughout a shared path, characterized by clear information, respect of time for listening, clinically appropriate answers, in an environment that patients perceive as empathetic. |
| ***10. Patient-reported-outcomes (PROs) specific for HS patients*** |
| 10.1 Ad hoc PROs assessments should be adopted systematically for all HS patients because they can accurately capture these patients’ lives, considering the huge impact of the disease on QoL. To date, (Hidradenitis Suppurativa quality of life) HiSQoL and Pain Index are the most validated and supported by scientific evidence. |
| ***11. Paucity of effective treatments currently available for HS to achieve optimal clinical outcomes and improvements in QoL*** |
| 11.1 Effects of current therapies often do not fulfill clinicians' and patients’ needs. Therefore, promotion and support of research for the development of new drugs (with clinical trials) and for optimization of existing treatments (with real-life studies) are fundamental. This would offer new hopes and solutions to patients. |
| ***12. Specific management of flares in HS*** |
| 12.1 A unanimous approach for the management of flares is not proposed by European and American guidelines currently available. The approach to flares depends on the expertise of dermatologists or surgeons, who treat the patient depending on his/her characteristics and ongoing therapies. |
| 12.2 A clear and common definition of HS flare and bacterial superinfection is necessary. In case of infection a targeted antibiotic therapy should be administered. |
| 12.3 In case of mild HS not being treated with systemic antibiotics, systemic antibiotics (e.g., tetracycline or clindamycin) can be started when flares occur. |
| 12.4 In case of moderate-severe HS being treated with systemic antibiotics, introduction of biologics can be considered when flares occur. Adalimumab is currently the only biologic refunded by the Italian Health Service for HS. |
| 12.5 In case of patients already on biological therapy, an increase in dose or frequency of administration (even temporary) could be considered when flares occur, although this approach (i.e., adalimumab 80 mg weekly) is now off-label. |
| 12.6 In case of frequent, long-lasting or non-responsive to aforementioned therapy flares, a switch to a different biologic drug could be considered, although most of them are currently off-label for HS. |
| 12.7 In case of localized flare in a single site, topical treatments could be considered, both intralesional infiltration of triamcinolone and local surgery, such as deroofing of abscessed lesions. |
| ***13. Combination of medical and surgical treatments for HS*** |
| - 1. Surgery is fundamental for specific HS-related conditions, such as abscesses, fistulas or scar tissue removal. A combined approach with drugs, such as biologics, is needed to control inflammation, enhancing benefits and reducing the risk of recurrency. |
| ***14. Smoking in HS patients*** |
| 14.1 The smoking habit represents a negative prognostic and therapy-response predictive factor. In addition, smoking is a well-known cardiovascular risk factor. |
| 14.2 Active and former smokers could have restrictions to access a novel class of drugs now under study for HS (i.e. JAK-inhibitors). |
| 14.3 The smoking habit should be discouraged in these patients, by enhancing the awareness about smoking consequences in HS and by joining stop smoking support programs. |
